# Case report of two patients with refractory HIV-related B-cell lymphoma treated with the monoclonal antibody glofitamab for secondary central nervous system involvement

**DOI:** 10.3389/fonc.2025.1699475

**Published:** 2026-02-17

**Authors:** Yueru Ji, Jing Gao, Tong Wei, Miaowang Hao, Weiwei Qin

**Affiliations:** Department of Hematology, Tangdu Hospital, The Fourth Military Medical University, Xi’an, China

**Keywords:** HIV-associated lymphoma, secondary central nervous system lymphoma, glofitamab, case report, effectiveness

## Abstract

Diffuse large B-cell lymphoma (DLBCL), the most common type of human immunodeficiency virus (HIV)-related lymphoma, is associated with a high risk of central nervous system (CNS) involvement. Studies have shown that CD20×CD3 bispecific antibodies have significant therapeutic efficacy in refractory B-cell lymphoma. Preliminary reports indicate that the monoclonal antibody glofitamab can penetrate the blood-brain barrier, inducing clinical responses in CNS DLBCL patients by activating T lymphocytes. This report presents preliminary clinical evidence on the use of glofitamab in HIV-positive patients with secondary CNS lymphoma.

## Introduction

Human immunodeficiency virus type 1 (HIV-1) can indirectly promote cancer through immune suppression; the associated mechanisms encompass the activation of latent tumor-associated viral infections, including Epstein-Barr virus (EBV)-associated B-cell lymphoma (1). Tumor-modulating viral factors affect tumor behavior through various pathways, including inducing inflammation in the tumor microenvironment (TME), inhibiting apoptosis, initiating angiogenesis, activating tumor cell signaling pathways to promote tumor growth, proliferation, and invasion, and altering tumor metabolism (2).

Diffuse large B-cell lymphoma (DLBCL) is the most common type of HIV-related lymphoma. Compared to sporadic DLBCL cases, HIV-DLBCL patients typically present with rapidly enlarging masses and systemic symptoms such as fever, night sweats, and weight loss (3). Moreover, HIV-DLBCL cases frequently exhibit extranodal lesions and are significantly associated with EBV, with relatively poorer prognosis (4). Few studies have compared the TME between sporadic DLBCL and HIV-related DLBCL, revealing significant differences: the TME of HIV-DLBCL exhibits features of increased angiogenesis, with significantly greater microvascular density than in sporadic cases; meanwhile, immune cells exhibit excessive proliferation and c-Myc rearrangements. Additionally, CD3+ T lymphocytes are markedly reduced in HIV-DLBCL patients, with significant decreases in helper T cells (CD4+) and regulatory T cells (FOXP3+), while CD8+ T cells abnormally accumulate in large numbers.

The pathological features of DLBCL include high tumor cell heterogeneity and resistance to chemotherapy, posing great challenges for diagnosis and treatment in HIV-infected individuals (5). Standard treatment protocols for DLBCL, including HIV-associated and central nervous system (CNS)-involved cases, have become increasingly aligned with general DLBCL management strategies. The current NCCN and major guidelines emphasize that patients with HIV-related DLBCL are treated similarly to HIV-negative patients, provided combination antiretroviral therapy (cART) is concurrently administered and careful attention is given to drug-drug interactions. The most commonly recommended regimens are R-CHOP (rituximab, cyclophosphamide, doxorubicin, vincristine, prednisone), DA-EPOCH-R (dose-adjusted etoposide, prednisone, vincristine, cyclophosphamide, doxorubicin, rituximab), and cART integration (6–8). CNS involvement in DLBCL, while uncommon, presents a major therapeutic challenge and influences both the regimen and the need for CNS prophylaxis. Systemic cytotoxic chemotherapy (such as R-CHOP or R-EPOCH) remains the backbone for patients with systemic and CNS involvement (9). According to recent NCCN Guidelines and international consensus, CNS prophylaxis should be considered only in patients with high-risk features: CNS-IPI (CNS International Prognostic Index) score of 4–6, involvement of ≥3 extranodal sites, or specific high-risk extranodal involvement (testicular, kidney, adrenal, or breast) (9, 10).

This article reports two cases of challenging HIV-related DLBCL, both of whom presented with CNS involvement and derived preliminary benefit through treatment with the monoclonal antibody glofitamab. The uniqueness of these cases lies in the demonstrated effectiveness of glofitamab in patients with CD4 immune cell deficiency while further supporting the drug’s therapeutic effects on CNS lesions. These findings provide important reference for differential diagnosis of HIV-related lymphoma, especially highlighting the clinical significance in the context of using CD3/CD20 dual-target antibody therapy based on the patient’s immune status.

## Case presentation

### Case 1

A 48-year-old male patient discovered a gradually enlarging mass in his left axilla in March 2023. A puncture biopsy assessed by immunohistochemistry confirmed a diagnosis of DLBCL (germinal center type), with the following marker detection results: LCA positive (+), CD20+, CD3-, CD19+, CD5-, CD30-, BCL-2-, BCL-6+, CD10+, CD56-, c-Myc+ (approximately 80%), MUM-1-, TIA-, Cyclin D1-, Ki67+ (90%), and EBER negative (–). A positron emission tomography (PET)/computed tomography (CT) scan showed a large, high-density shadow in the left intermuscular space and left axilla, with significantly increased glucose metabolism (SUVmax 26), indicating involvement of the left submandibular lymph nodes. The patient had a prior history of HIV-related lymphoma (DLBCL, germinal center type, stage II B). Targeted next-generation sequencing analysis revealed: c-Myc-IgH fusion (36.95%); TP53 mutation sites, c.731G>A (p.G244D, 50.75%) and c.701A>G (p.Y234C, 42.84%). Additionally, peripheral blood circulating tumor DNA (ctDNA) testing revealed a DNMT3A mutation (exon8, c.942G>A, p.W314*, 0.8%; exon19, c.2186G>A, p.R729Q, 2.8%).

### Treatment process

The patient received lymphoma treatment as shown in **Table 1**. The R-CDOP (rituximab, cyclophosphamide, liposomal doxorubicin, vincristine, and prednisone) cycle in April 2023 led to disease progression. Two cycles of R-MAE in May 2023 first led to a partial response, followed by progression. Two cycles of R-GMD plus orelabrutinib did not have a significant response. Three cycles of dexamethasone, sintilimab, and GEMOX (gemcitabine and oxaliplatin) in September 2023 achieved a partial response. Left axillary radiotherapy in December 2023 also achieved a partial response.

**Table 1:** Lymphoma treatment process in Case 1.

Regimen	Drug and dosage	Efficacy evaluation
2023.04.16 R-CDOP×1	Rituximab 375 mg/m^2^, d0, Cyclophosphamide 750 mg/m^2^ d1, Doxorubicin hydrochloride liposome 32 mg/m^2^ d1, Vincristine 2 mg d1, Prednisone acetate 1 mg/kg d1-5	PD
2023.05.04/2023.06.02 R-MAE×2	Rituximab 775 mg/m^2^ d0, Mitoxantrone liposome 21 mg/m^2^ d1, Etoposide 200 mg d1, 150 mg d2-3 (270 mg/m^2^); Cytarabine 1.87 g/m^2^ q12, d2-3; Dexamethasone 30 mg d1-3	PR-PD
2023.07.07/2023.08.04 R-GMD + Orelabrutinib×2	Rituximab 375 mg/m^2^ d0; Gemcitabine 1g /m^2^ d1,8; Mitoantrone liposome 21 mg d1; Dexamethasone 40 mg d1-4; Orelabrutinib 150 mg/day d1-21	PD
2023.09.19/2023.10.15/2023.11.09 Dexamethasone + Sintilimab + GEMOX×3	Gemcitabine 10 mg subcutaneously d1-5; Gemcitabine 0.9 g/m^2^ d1,8; Oxaliplatin 90 mg/m^2^ d2; Sintilimab 200 mg d6	PR
2023.12.20 Left axillary wild radiotherapy	45 Gy	PR

**Figure 1:**
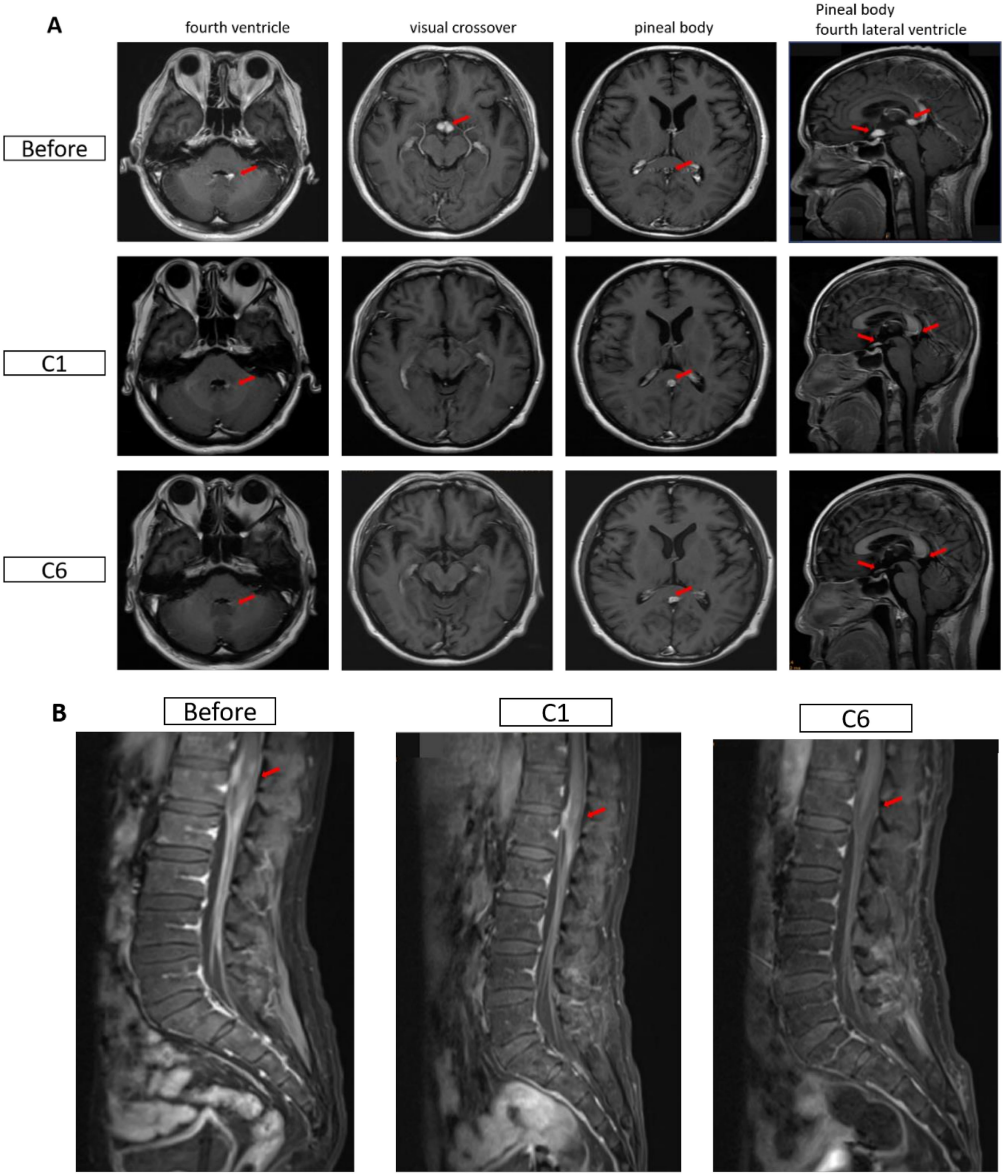
Magnetic resonance imaging (MRI) images of the head in both patients. **(A)** Changes in cranial MRI before and after treatment for Case 1. Before: April 7, 2024; C1: May 6, 2024; C6: August 15, 2024. **(B)** Changes in the conus and cauda equina regions before and after treatment for Case 2. Before: May 25, 2024; C1: June 26, 2024; C6: November 14, 2024. The red arrows indicate the lesions.

One month after radiotherapy, the patient could not undergo treatment due to influenza A virus infection, which was managed using baloxavir marboxil. In early April 2024, the patient had an increased axillary mass again, accompanied by headaches, nausea, vomiting, discomfort, and blurred vision on the left side. Head magnetic resonance imaging (MRI) in April 2024 suggested abnormal enhancement foci in the bilateral optic chiasm, pineal region, and the left wall of the fourth ventricle, indicating potential lymphoma invasion (**Figure 1A**). The diagnosis was HIV-related secondary DLBCL of the central nervous system. Blood routine tests indicated a platelet (PLT) count of 32 × 10^9^/L and normal white blood cell and hemoglobin levels. B lymphocyte count was 12/µl. Cytokine test results: IL-6, 58.96 pg/ml; IFN-γ, 64.79 pg/ml. Lactate dehydrogenase (LDH) was 1088 U/L, C-reactive protein (CRP) was 66.4 mg/L, and CD4 cell count was 255 cells/μL. The subsequent treatment and T cell data of the patient are shown in **Table 2**.

**Table 2:** Subsequent treatment and T cell data of Case 1.

Time/Period	Drug and dosage	Efficacy	T-Cell count
2024.4.8 C1	Zuberitamab 600 mg D0; Decitabine 10 mg, D1-5; Tislelizumab 200 mg, D6; Glofitamab 2.5 mg, D8; 10 mg, D15	PR	CD3-T 1017/ul; CD4-T 188/ul; CD8-T 808/ul
2024.5.6/2024.5.28/2024.6.17/2024.7.9/2024.7.30 C2-6	Glofitamab 30 mg, D1 Orelabrutinib 150 mg, D1-21	PR	CD3-T 1028/ul; CD4-T 305/ul; CD8-T 741/ul

**Figure 2:**
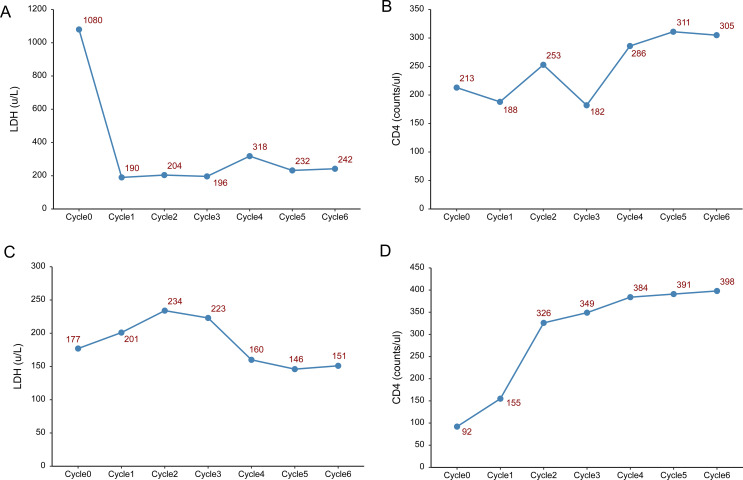
Changes in lactate dehydrogenase (LDH) and lymphocyte count. **(A–C)** Changes in LDH, CD4 and CD8 lymphocyte count before each treatment cycle in Case 1. **(D–F)** Changes in LDH, CD4 and CD8 lymphocyte count before each treatment cycle in Case 2.

During treatment, the patient developed a fever 1–2 days after the glofitamab infusion, with a maximum temperature of 38.3°C, and no specific treatment was given. Lactate dehydrogenase decreased sharply after the first cycle, while CD4+ and CD8+ cells showed a progressive increase during treatment (**Figures 2A–C**). HIV viral load remained undetectable. On August 15, 2024, the mass in the left axilla increased again, and new subcutaneous masses appeared under the left scapula as well as in multiple areas of the left anterior chest. No enlargement of the lesions was observed by cranial MRI. Pathological examination of the left axillary mass suggested DLBCL, indicating disease progression. Subsequently, the patient was administered two cycles of polatuzumab vedotin combined with the GDP chemotherapy regimen (gemcitabine, dexamethasone, and cisplatin), which was ineffective. The patient died in November 2024 due to treatment failure.

### Case 2

A 62-year-old male patient experienced swelling and pain in the right gum in December 2023, with symptoms progressively worsening. He underwent excision of the right gum mass, and pathological immunohistochemical results showed CD20+, CD3-, CD30-, Ki67>90%, CD38+, CD34-, PAX-5+, CD56-, BCL-2-, BCL-6+ (70%), CD10+, and C-MYC (70%), indicating DLBCL (germinal center type). Concurrently, HIV was detected. PET/CT examination suggested involvement of bilateral alveola, left maxillary sinus, right temporomandibular joint, bilateral hilum of lungs, hepatic hilum, retroperitoneum, mesentery, localized pleura of both lungs, left outer lobe of the liver, subcapsular region of the right kidney, bilateral adrenal glands, right 10th rib and left 5th rib; nodular metabolic increase in the gastric wall at the cardia area, with a high possibility of lymphoma involvement; nodular glucose metabolism increase in the bilateral lateral zones of the prostate and the left seminal vesicle, suspected of involvement. Peripheral blood ctDNA genetic mutation analysis revealed a PPM1D mutation (c.1654c>t, p.R552* mutation frequency 13.10%) with a final diagnosis of HIV-related lymphoma (DLBCL, germinal center type, IVB).

### Treatment process

The patient achieved complete remission after receiving four cycles of R-CODP (started in December 2023) but developed symptoms of double vision, lower limb numbness, and difficulty urinating within 2 months (Table 3). The patient received a combination of rituximab, methotrexate, thiotepa, and orelabrutinib. MRI scans of the head and lumbar spine in May 2024 showed abnormal enhancement of the conus medullaris and cauda equina, initially considered due to lymphoma infiltration (**Figure 1B**). The final diagnosis was refractory HIV-related lymphoma, associated with secondary central nervous system invasion. After six cycles of single-agent treatment with glofitamab, the patient declined further treatment for personal reasons. During treatment, LDH increased and then decreased, while CD4+ steadily increased. CD8+ cells showed a steep increase followed by a plateau (**Figures 2D–F**). HIV viral load remained undetectable. As of December 2024, the response assessment showed the patient had achieved partial remission.

**Table 3:** Treatment process in Case 2.

Regimen	Drug and dosage	Efficacy	T-Cell count
2023.12.29/2024.1.30/2024.2.29/2024.03.29 C1-4: R-CDOP	Rituximab 375mg/m^2^, d0, Cyclophosphamide 750mg/m^2^d1, Doxorubicin hydrochloride liposome 30mg/m^2^ d1, Vincristine 2 mg d1, Prednisone acetate 50mg/m2 d1-5	CR	CD3-T 1250/ul; CD4-T 213/ul; CD8-T 963/ul
The disease progressed in 2 months			
2024.05.11 C1: R+MTX+ thiotepa+ Orelabrutinib	Rituximab 375mg/m^2^ , d0; Methotrexate 3g/m^2^ for maintenance 3h, d1; Thiotepa 30mg/m^2^ d2; Dexamethasone 10 mg d1-2 Orelabrutinib 150 mg, D1-21	SD	CD3-T 875/ul; CD4-T 92/ul; CD8-T 745/ul
2024.6.01/2024.6.26/2024.7.24/2024.8.28/2024.9.26 C1-6: glofitamab	C1: Glofitamab 2.5 mg, D1; 10 mg, D8 C2-6: Glofitamab 30 mg, D1	PR	CD3-T 2005/ul; CD4-T 398/ul; CD8-T 1437/ul

### Adverse events in the two patients after treatment of glofitamab

The adverse events (AEs) for both patients were grade 1 cytokine release syndrome (CRS) reactions, occurring within 1–2 days post-infusion and during the first three treatment cycles (C1-C3) of glofitamab. To prevent CRS reactions, prophylactic doses of acetaminophen 1000 mg, dexamethasone 20 mg, and diphenhydramine 50 mg were administered before treatment during the first three cycles (C1-C3). For the subsequent three cycles (C4-6), acetaminophen 1000 mg and diphenhydramine 50 mg were administered. No specific treatment was administered following the occurrence of a CRS reaction.

### HIV management considerations for both patients

The two patients had undetectable HIV viral loads before antitumor therapy and were on regular oral antiretroviral treatment with Biktarvy (a fixed-dose once-daily triple-drug regimen of bictegravir, emtricitabine plus tenofovir alafenamide). They concurrently took acyclovir and co-trimoxazole to prevent viral and *Pneumocystis jirovecii* infections.

## Discussion

This article reports two cases of HIV-related lymphoma patients, both of whom had confirmed DLBCL through immunohistochemistry. Both patients had secondary central nervous system involvement, with clinical treatment showing refractory disease. The complex association between HIV infection and DLBCL, along with the resulting clinical practice challenges, warrants in-depth discussion. Epidemiological data indicate that HIV-infected individuals have a significantly increased risk of developing lymphoma, particularly DLBCL, which is one of the main types of HIV-related lymphoma, displaying significant pathological features and clinical manifestations different from the general population (3). This article discusses the treatment of HIV-related B-cell lymphoma with central nervous system involvement using glofitamab, providing clinical evidence for this treatment approach in HIV-related B-cell lymphoma.

The mechanisms underlying the occurrence of HIV-related lymphoma are extremely complex, involving various factors, e.g., immunosuppression, chronic inflammation, co-infection with other oncogenic viruses (such as EBV), and adverse lifestyle habits (11). Research has shown that certain proteins of HIV (such as p17 and Tat protein) are closely related to the occurrence and development of lymphoma (12, 13). Additionally, the immunological imbalance caused by HIV infection reduces the body’s ability to clear oncogenic viruses like EBV, thereby significantly increasing the risk of lymphoma (13, 14).

At the genetic level, lymphomas in HIV-infected individuals exhibit unique mutation characteristics, e.g., high expression of c-MYC and mutations in TP53. The protein encoded by the c-MYC gene is a key transcription factor that regulates the cell cycle, proliferation, metabolism, and apoptosis (15). Various studies have indicated that increased c-MYC protein expression and MYC gene copy number may influence disease progression and prognosis (11). A previous study showed significantly elevated c-MYC expression in HIV-related DLBCL patients (16), and c-MYC protein expression rates in the two patients discussed in this article were 80% and 70%, respectively, with 36.95% c-MYC-IgH rearrangement detected in Case 1. The abnormal activation of c- MYC usually results from chromosomal translocations, such as t(8;14), which place c-MYC adjacent to immunoglobulin genes (IGH or IGK/IGL), leading to sustained overexpression of c-MYC (17). Approximately 80% of Burkitt lymphoma cases exhibit MYC-IGH translocations (18), but MYC rearrangements can also be detected by fluorescence *in situ* hybridization (FISH) in about 12% of DLBCL cases (19). Furthermore, Chapman et al. through FISH analysis of HIV-DLBCL, identified MYC translocations in 9 of 16 cases (56%), a rate markedly higher than that in the general DLBCL population (20). Mutations in TP53 result in the functional loss of the p53 protein, thereby impairing cell cycle regulation, DNA repair, and apoptosis, and are correlated with a poor prognosis in various tumors, including DLBCL (21). Furthermore, several studies have reported a prevalence of TP53 mutations in HIV-associated DLBCL (22, 23). Moreover, it has been reported that DLBCL patients with concurrent TP53 mutations and c-MYC rearrangements exhibit an even poorer prognosis (24). In case 2 of this study, a PPM1D mutation was detected in the ctDNA. Mutation in PPM1D inhibits p53-mediated transcription and apoptosis by suppressing p53 phosphorylation (25). Therefore, those molecular characteristics may suggest the aggressive and refractory nature of HIV-associated DLBCL. Clonal hematopoiesis of indeterminate potential (CHIP) specifically refers to clonal hematopoiesis with somatic mutations in hematopoietic driver genes exhibiting a variant allele frequency (VAF) of at least 2%, occurring in the absence of diagnosed hematologic disease or cytopenia. The DNMT3A mutation is one of the most common mutations in CHIP (26). In Case 1, this mutation exhibits a low VAF and is not typical of lymphoma driver genes. Therefore, it likely represents a coexisting, lymphoma-unrelated CHIP.

In summary, HIV infection triggers immune dysfunction and chronic inflammation, decreasing the body’s ability to clear carcinogenic factors; simultaneously, abnormalities in genes such as c-MYC, and TP53 further lead to uncontrolled cell proliferation and inhibited apoptosis. These factors collectively contribute to the occurrence of lymphoma. Both patients showed chemotherapy resistance and short-term relapse during previous treatments, along with significant genomic abnormalities, reflecting the high-risk genetic landscape and clinical characteristics of HIV-related lymphoma.

Previous retrospective studies have reported significant risk factors for HIV-related DLBCL, including CD4 cell count <150/mm³ (27), IPI score of 3-5 (16), and high IL-6 levels (28), all associated with poor survival. A domestic case study included 273 newly diagnosed HIV-related DLBCL patients, among whom 63.7% had CD4 cell counts below 200/mm³, with a median two-year overall survival rate (OS) of 58.0%. Multivariate analysis showed that age ≥60 years, high IPI score, B symptoms, elevated LDH, and insufficient chemotherapy cycles (<4) were independent risk factors for poor prognosis. IPI scores at central relapse for the two patients in this article were 3 points (Case 1) and 4 points (Case 2), both associated with adverse prognostic features such as B symptoms. Both patients experienced elevated LDH during the secondary central involvement and disease progression, which gradually decreased with glofitamab treatment. Monitoring CD4-T cell counts in these patients revealed T cell proliferation with dual-antibody therapy.

Changes in peripheral blood CD8+ T cell counts, as well as their recruitment and activation within the tumor microenvironment, are key aspects of the immune landscape during lymphoma treatment. In B-cell lymphomas, and specifically in DLBCL, peripheral blood typically exhibits an expansion of CD8+ T-cell populations in response to immunomodulatory or T-cell-engaging therapies, such as bispecific antibodies or checkpoint blockade. These CD8+ T cells often exhibit activated phenotypes, as indicated by expression of effector and memory markers and increased production of cytotoxic cytokines, such as interferon gamma, following stimulation (29, 30). Within the tumor microenvironment, recruited CD8+ T-cells are pivotal in mediating antitumor responses. Research indicates that, particularly in DLBCL, the majority of infiltrating CD8+ T cells are highly activated and not exhausted, maintaining robust effector functions despite expressing regulatory receptors such as PD-1, TIM-3, or CTLA-4. Bispecific antibody therapies further promote the expansion and recruitment of both chimeric antigen receptor-positive and bystander CD8+ T-cells from the periphery to tumor sites, augmenting immune-mediated tumor cell lysis and potentially overcoming local immune suppression (30, 31). Overall, dynamic monitoring of peripheral and tumor-infiltrating CD8+ T-cell populations provides valuable insight into treatment response and the functional state of antitumor immunity in B-cell lymphomas.

About 40% of non-Hodgkin lymphoma patients face the dilemma of refractory relapse, with 4-6% experiencing central relapse (32). Treatment for secondary central nervous system lymphoma usually involves high-dose chemotherapy regimens capable of penetrating the blood-brain barrier, but the median survival time is only about 6 months (33, 34). The incidence of central involvement among HIV-infected individuals is substantially high. A retrospective review of 886 patients revealed that 13% had CNS involvement, with a median OS of only 1.6 months post-central involvement (35). The treatment of secondary central nervous system lymphoma remains challenging, and immunotherapy has been attempted in this disease. Research shows that chimeric antigen receptor T cell (CAR-T) therapy has marked efficacy against central nervous system cancers (36). The complete response rate for the CD3-CD20 dual-targeted glofitamab in refractory DLBCL was 39%, with an objective response rate of 52% and 78% of complete responses lasting at least 12 months, while the progression-free survival rate was 37% (37). However, due to CD4 cells’ functional deficiencies in HIV patients, the application of autologous CAR-T cell therapy in such patients is limited. Godfrey et al. treated four patients with chemotherapy and CAR-T treatment failures for secondary central nervous system lymphoma using CD3-CD20 dual-target glofitamab, which had high efficacy in three cases. This study further supports the notion that glofitamab can partially penetrate the blood-brain barrier and induce clinical and imaging improvements in patients with central lymphoma. It is efficacious at extremely low concentrations in cerebrospinal fluid (38). Still, no lumbar punctures to obtain cerebrospinal fluid for examination were performed in the two cases reported here, either at the time of CNS recurrence diagnosis or during glofitamab treatment, as the diagnosis of CNS involvement was definitively established based on characteristic MRI findings.

The patients reported in this article received glofitamab treatment following central involvement. Given that glofitamab can cross the blood-brain barrier and that CD3 T lymphocyte counts in both patients remained within the relative normal range, this treatment regimen was adopted. For Case 1, due to the heavy tumor burden and the potential for severe CRS reactions, especially immune effector cell-associated neurotoxicity syndrome (ICANS), the treatment initially employed decitabine and tislelizumab in the first cycle to reduce the tumor load and maintain immune function. Decitabine, a hypomethylating agent, can upregulate tumor-associated antigens, improve antigen presentation, and reverse tumor immune evasion. It has been shown to sensitize tumor cells to immune attack and enhance the tumor microenvironment for subsequent immunotherapies (39). Tislelizumab is an anti-PD-1 monoclonal antibody that blocks inhibitory signaling in T cells, reinvigorating antitumor immune responses. When combined with decitabine, emerging studies demonstrate reversal of PD-1 resistance, allowing deeper immune infiltration and cytotoxic activity, even in heavily pretreated or immunosuppressed settings (40). The enhanced immune microenvironment induced by decitabine and tislelizumab may prime the host immune system and tumor niche to respond more robustly to subsequent CD20- or CD19-targeting agents, such as glofitamab, by maximizing immune recognition and minimizing initial tumor burden, thereby theoretically reducing the risk of overwhelming cytokine release (41). Treatment outcome analysis showed that both patients achieved partial remission, with Case 1’s remission lasting 4 months before death due to disease progression, possibly related to the low CD4 cell count and function reported in HIV patients. Therefore, it is speculated that, based on adequate infection prevention, combination therapy with glofitamab (e.g., PD-1 monoclonal antibodies or chemotherapy combinations) could provide more treatment options for patients with HIV-related lymphoma and secondary central involvement. Currently, Case 2 remains in continuous remission. In terms of adverse reactions, both patients only experienced grade 1 CRS reactions, requiring no special management, which may be related to their immunocompromised status. However, the risk of CRS, especially ICANS, remains a significant concern for immunocompetent patients.

Liquid biopsy, particularly through the analysis of ctDNA, is rapidly emerging as a transformative tool in the management of B-cell lymphomas, complementing and often surpassing conventional tissue biopsy in clinical utility. Almasri et al. (42) provide a comprehensive overview of liquid biopsy’s applications, emphasizing its role in non-invasive genotyping, tracking molecular response dynamics, and identifying prognostic and predictive biomarkers in both B- and T-cell lymphomas. For DLBCL, ctDNA enables precise detection of hallmark mutations, facilitates molecular subtyping, and enables molecular clustering, each critical for risk stratification and therapeutic targeting (42). Molecular clustering via liquid biopsy holds particular promise for HIV-related lymphomas, a population characterized by marked molecular and clinical heterogeneity. In patients treated with novel bispecific antibodies such as glofitamab, this approach could support real-time monitoring of clonal evolution and minimal residual disease, inform adaptive treatment strategies, and help individualize therapy, ushering in a new era of precision medicine. Such stratification is especially valuable in the context of immunotherapy, where early detection of emerging resistant clones or molecular subtypes could guide timely intervention or combination therapies, ultimately improving outcomes for this challenging subgroup (42, 43).

Selecting glofitamab over CAR-T cell therapy for HIV-positive lymphoma patients was grounded in both immunologic and practical considerations unique to this population. Indeed, HIV-positive patients, particularly those with a history of profound CD4+ T cell depletion, may have compromised T-cell numbers and function even on ART, which presents challenges for autologous CAR-T therapy manufacture and expansion. While CD8+ cytotoxic T cells play a crucial role in CAR-T efficacy, durable clinical responses often rely on a robust CD4+ compartment for sustained support and proliferation of CAR-T cell products. Additionally, HIV-infected CD4+ T cells can harbor latent virus, raising the risk that manufactured CAR-T products could be contaminated with HIV or be less functional (44, 45). CAR-T cell therapy requires a lengthy, highly specialized manufacturing process, starting with the collection of adequate numbers of functional T cells via apheresis. HIV-positive patients, especially those with low CD4+ counts or prior intensive therapy, may not yield sufficient quality T cells, leading to a risk of manufacturing failure or delays. Glofitamab, as an off-the-shelf bispecific antibody, overcomes this bottleneck by enabling immediate treatment initiation, which is critical for patients with aggressive disease or rapidly progressing relapsed/refractory lymphoma (44, 46, 47). Although emerging data show that HIV-positive lymphoma patients can tolerate CAR-T therapy comparably to HIV-negative counterparts when viral load is controlled, there remains an elevated risk of treatment-related infectious complications due to underlying immune deficiency, especially when CAR-T-induced cytopenias are layered atop HIV-related immunosuppression. Glofitamab has a favorable, manageable safety profile, particularly regarding cytopenias and infectious risks, making it highly suitable for heavily pretreated or immunocompromised patients. Its adverse event profile, including CRS and neurotoxicity, is generally lower grade and manageable with careful monitoring and premedication (46–48).

Furthermore, according to the NCCN, high-dose chemotherapy followed by autologous stem cell transplantation (ASCT) is a standard option for relapsed/refractory DLBCL in chemosensitive, fit patients with no active CNS involvement (8). Here, the patients did not undergo ASCT due to factors such as chemoresistant/refractory disease, significant HIV-associated immunosuppression, risk of infectious complications, or poor performance status, which were clear contraindications per guidelines.​ Second, CNS prophylaxis with HD-MTX and/or intrathecal (IT) chemotherapy is recommended for high-risk patients (e.g., certain extranodal sites, high CNS-IPI score). Administration can occur during or after induction therapy, though recent studies question the benefit of routine prophylaxis (8).​ The omission of HD-MTX or IT chemotherapy was justified by the patients’ CNS relapse risk, performance status, renal function, or logistical and toxicity concerns. Third, CAR-T therapy is recommended for relapsed/refractory DLBCL patients who have failed ≥2 lines of systemic therapy and are ASCT-ineligible or have failed prior ASCT. Suitability requires adequate T-cell fitness and no active severe infections (8). In the two cases, CAR-T was not chosen, concordant with recognized issues in HIV-positive patients: diminished T-cell quantity/fitness (especially CD4+ deficiency), increased risk of infectious or immune complications, and a high risk of CAR-T manufacturing failure. Glofitamab was favored due to immediate availability, lower manufacturing barriers, and a safer profile in immunocompromised patients (44, 46). In addition, glofitamab was selected because both patients developed resistance to multiple standard therapies. Although polatuzumab vedotin + GDP is also an option for relapsed/refractory DLBCL, the patients’ prior treatment history and potential hematologic toxicity were considered.

The pivotal phase II study by Dickinson et al. (31) demonstrated impressive efficacy and a manageable safety profile of glofitamab in patients with relapsed or refractory DLBCL. However, it is important to emphasize that the trial specifically excluded individuals with active CNS lymphoma and those with HIV infection, thereby limiting the generalizability of the findings to these higher-risk populations. This exclusion highlights the need for real-world evidence regarding glofitamab’s feasibility in HIV-positive DLBCL patients with secondary CNS involvement, such as the cases under discussion. While extrapolating from the pivotal study must be approached with caution, our clinical experience suggests that glofitamab may maintain a favorable effectiveness and a manageable toxicity profile even in this particularly challenging subset. Furthermore, CRS was the most common adverse event reported in Dickinson et al.’s trial, consistent with observations in the present cases, in which CRS emerged as the primary treatment-related complication. This similarity further supports the relevance of trial safety data to the real-world context, suggesting continuity in adverse event profiles while underscoring the necessity for vigilant monitoring in immunocompromised or CNS-involved patients (49).

In summary, secondary CNS lymphoma is a high-risk, therapeutically challenging population characterized by poor outcomes despite advances in intensive chemotherapies and newer cellular or targeted therapies. Evidence gaps persist regarding optimal therapy, prevention, and integration of precision medicine tools, underscoring the importance of ongoing research and tailored, risk-adapted management (50, 51).

## Conclusion

Treatment with glofitamab showed preliminary benefits in two patients with HIV-related lymphoma and secondary CNS involvement, although the effectiveness was slightly less than that observed in refractory non-HIV lymphoma patients, primarily attributed to immune dysfunction in these patients, particularly insufficient immune cell levels. Nevertheless, this treatment regimen demonstrated certain feasibility, especially in securing valuable time for subsequent autologous hematopoietic stem cell transplantation and other therapies.

## Data Availability

The original contributions presented in the study are included in the article/supplementary material. Further inquiries can be directed to the corresponding author.
